# Effects of cell-cycle-dependent expression on random fluctuations in protein levels

**DOI:** 10.1098/rsos.160578

**Published:** 2016-12-07

**Authors:** Mohammad Soltani, Abhyudai Singh

**Affiliations:** 1 Department of Electrical and Computer Engineering, University of Delaware, Newark, DE, USA; 2 Department of Mathematical Sciences, University of Delaware, Newark, DE, USA; 3 Department of Biomedical Engineering, University of Delaware, Newark, DE, USA

**Keywords:** cell-to-cell variability, moment dynamics, noise decomposition, stochastic cell-cycle times, cell division

## Abstract

Expression of many genes varies as a cell transitions through different cell-cycle stages. How coupling between stochastic expression and cell cycle impacts cell-to-cell variability (noise) in the level of protein is not well understood. We analyse a model where a stable protein is synthesized in random bursts, and the frequency with which bursts occur varies within the cell cycle. Formulae quantifying the extent of fluctuations in the protein copy number are derived and decomposed into components arising from the cell cycle and stochastic processes. The latter stochastic component represents contributions from bursty expression and errors incurred during partitioning of molecules between daughter cells. These formulae reveal an interesting trade-off: cell-cycle dependencies that amplify the noise contribution from bursty expression also attenuate the contribution from partitioning errors. We investigate the existence of optimum strategies for coupling expression to the cell cycle that minimize the stochastic component. Intriguingly, results show that a zero production rate throughout the cell cycle, with expression only occurring just before cell division, minimizes noise from bursty expression for a fixed mean protein level. By contrast, the optimal strategy in the case of partitioning errors is to make the protein just after cell division. We provide examples of regulatory proteins that are expressed only towards the end of the cell cycle, and argue that such strategies enhance robustness of cell-cycle decisions to the intrinsic stochasticity of gene expression.

## 1. Introduction

Advances in experimental technologies over the last decade have provided important insights into gene expression at a single-molecule and single-cell resolution. An important (but not surprising) revelation is the stochastic expression of genes inside individual cells across different organisms [1–11]. In many cases, stochastic expression is characterized by random burst-like synthesis of gene products during transcription and translation. At the transcriptional level, promoters randomly switch to an active state, producing a burst of RNAs before becoming inactive [12–17]. At the translational level, a relatively unstable mRNA degrades after synthesizing a burst of protein molecules [18–21]. Bursty expression drives intercellular variability in gene product levels across isogenic cells, significantly impacting biological pathways and phenotypes [22–29].

**Figure 1.**
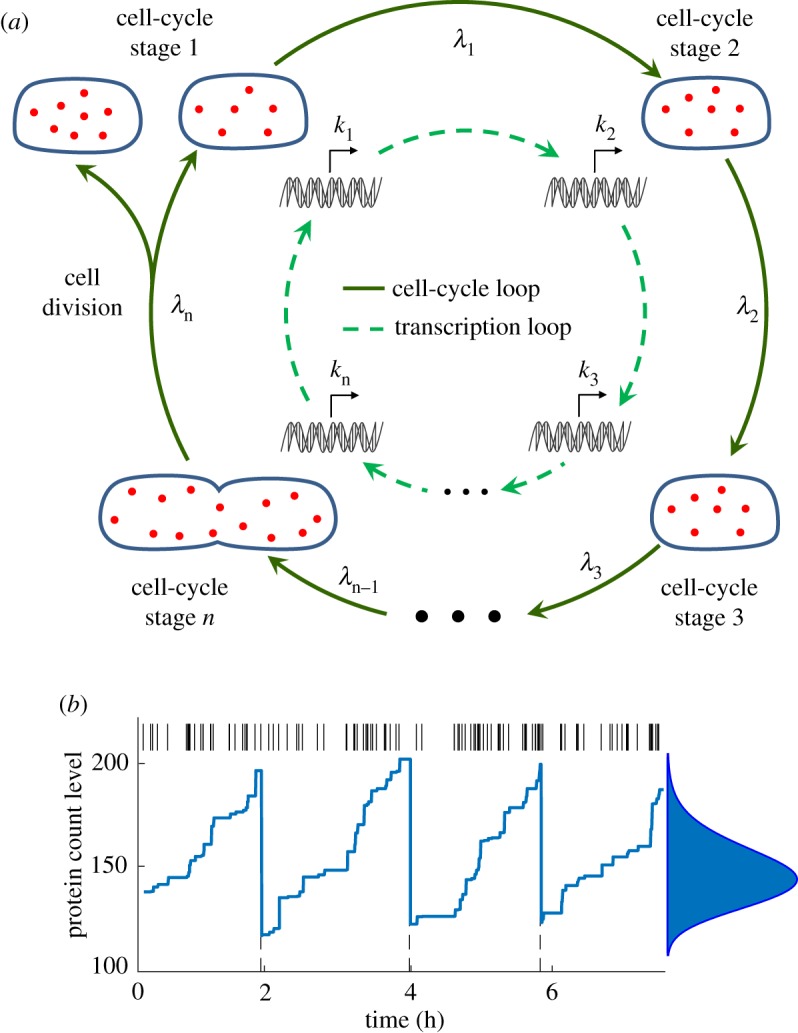
Coupling cell cycle to gene expression. (*a*) The outer loop shows an individual cell from birth to division passing through cell-cycle stages *C*_1_,*C*_2_,…,*C*_*n*_, with transition rates between stages given by λ_*i*_, *i*∈{1,2,…,*n*}. The cell is born in stage *C*_1_ and division is initiated in *C*_*n*_. The inner loop (transcriptional cycle) represents the rate at which protein expression bursts occur and is given by *k*_*i*_ in cell-cycle stage *C*_*i*_. (*b*) Representative trajectory of the protein level in an individual cell through multiple cell cycles (dashed lines). In this case, the transcription rate is assumed to double at the cell-cycle midpoint due to replication-associated increase in gene dosage. The spike train above represents the firing times of burst events. Steady-state distribution of the protein copy numbers obtained from running a large number of Monte Carlo simulations is shown on the right. The cell cycle was modelled by choosing *n*=20 stages with equal transition rates between. It means that noise in cell-cycle time is $CV_{T}^{2}=0.05$ [35]. Protein expression was assumed to occur in geometric bursts with 〈*B*〉=4 [36] and molecules were partitioned between daughter cells based on a binomial distribution. Cell-cycle time is selected to be 〈*T*〉=2 *h* as measured in slow-growing cells [37].

Mathematical models have played a key role in predicting the impact of bursty expression on noise in the level of a given protein. However, these studies have primarily relied on models where synthesis rates are assumed to be constant and invariant of cell-cycle processes. While such an assumption is clearly violated for cell-cycle-regulated genes [30], replication-associate changes in gene dosage can alter expression parameters genome wide [31–34]. It is not clear how such cell-cycle-dependent expression affects the stochastic dynamics of protein levels in single cells. To systematically investigate this question, we formulate a model where a cell passes through multiple cell-cycle stages from birth to division. Cell cycle is coupled to bursty expression of a stable protein and the rate at which bursts occur depends arbitrarily on the cell-cycle stage (figure 1). In addition to stochastic expression in bursts, the model incorporates other physiological noise sources, such as variability in the duration of cell-cycle times and random partitioning of molecules between daughter cells at the time of division [38–46].

In the proposed model, some cell-to-cell variability or noise in the protein level is simply a result of cells being in different cell-cycle stages (i.e. asynchronous population). We illustrate a novel approach that takes into account such cell-cycle effects, and quantifies the noise contribution just from bursty expression and partitioning errors. Formulae obtained using this approach reveal that cell-cycle-dependent expression considerably alters noise, always affecting contributions from bursty expression and partitioning errors in opposite ways. Intriguingly, our results show existence of optimal strategies to synthesize a protein within the cell cycle that minimize noise contributions for a fixed mean protein level. For example, the noise contribution from bursty expression is minimal when the protein is synthesized only towards the end of cell cycle. We discuss intuitive reasoning behind these optimal strategies, and provide examples of proteins that are expressed in this manner to enhance fidelity of cell-cycle decisions.

## 2. Model coupling cell cycle to gene expression

We adopt a phenomenological approach to model the cell cycle and divide it into *n* stages *C*_1_,*C*_2_,…,*C*_*n*_. A newborn cell is in stage *C*_1_ and transitions from *C*_*i*_ to *C*_*i*+1_ with rate λ_*i*_. In stage *C*_*n*_, cell division is initiated with rate λ_*n*_, and upon division the cell returns to *C*_1_. In the stochastic formulation of this model, the cell resides in stage *C*_*i*_ for an exponentially distributed time interval with mean 1/λ_*i*_, and cell-cycle duration is a sum of *n* independent, but not necessarily identical, exponential random variables. These stages can be mathematically characterized by Bernoulli processes *c*_1_(*t*),*c*_2_(*t*),…,*c*_*n*_(*t*), where *c*_*i*_(*t*)=1 when the cell is in stage *C*_*i*_ and *c*_*i*_(*t*)=0 otherwise. Based on the model structure, these processes satisfy

2.1 $$\sum_{i=1}^{n}c_{i}(t)=1,\;c_{i}(t)c_{j}(t)=0\;\mathrm{for}\;i\neq j.$$

The latter equality results from the fact that only one of the *c*_*i*_ can be equal to 1 at any given time. In addition, since *c*_*i*_ takes values in {0,1}

2.2 $$\langle c_{i}^{m}\rangle =\langle c_{i}\rangle ,\;m\in \{1,2,\ldots \},$$

where the symbol 〈 〉 denotes the expected value. Next, we describe the coupling between the cell cycle and stochastic expression models.

We assume that gene-expression bursts occur at a Poisson rate *k*_*i*_ in cell-cycle stage *C*_*i*_. Using the above-defined Bernoulli processes, the burst arrival rate can be compactly written as $\sum_{i=1}^{n}k_{i}c_{i}(t)$. Let *x*(*t*) denote the number of protein molecules in a single cell at time *t*. Then, whenever burst events occur, the protein level is reset as

2.3 x(t)↦x(t)+B,

where the protein burst size *B*∈{0,1,2,…} is a random variable independently drawn from an arbitrary distribution, and reflects the net contribution of transcriptional and translational bursting. As is true for most proteins in Escherichia coli and Saccharomyces cerevisiae, we assume a stable protein without any active degradation between burst events [36,47,48]. At the time of cell division (as dictated by the cell-cycle model), the protein molecules are randomly partitioned between daughters. This corresponds to the following reset that is activated during division:

2.4 $$x(t)\mapsto x_{+}(t),$$

where the mean and variance of *x*_+_ (level just after division) conditioned on *x* (level just before division) are given by

2.5 $$\langle x_{+}\;|\;x\rangle =\frac{x}{2}\;\mathrm{and}\;\langle x_{+}^{2}\;|\;x\rangle -{\left\langle x_{+}\right\rangle }^{2}=\frac{\alpha x}{4},$$

respectively. The first equation in (2.5) shows that the number of molecules is approximately halved during division, i.e. on average each daughter cell inherits half the proteins of the mother cell. The second equation quantifies the stochasticity in the partitioning process through the parameter *α*≥0. It turns out that alteration in *α* is a convenient approach to realize different partitioning scenarios. For example, the ideal case of deterministic partitioning (or zero partitioning errors) corresponds to *α*=0, where *x*_+_(*t*)=*x*(*t*)/2 with probability one. Binomial partitioning, where each molecule has an equal chance of ending up in one of the two daughter cells, is given by *α*=1 [49–51]. In many cases, additional control mechanisms (such as molecules pushing each other to opposite poles of the cell) are used to suppress stochasticity in the partitioning process [38]. This leads to sub-binomial partitioning errors that can be phenomenologically modelled by selecting *α*<1. Finally, values of *α*>1 represent additional errors in partitioning that arise when protein molecules form multimers, or reside in organelles that are themselves subject to binomial partitioning [52,53]. It is important to point out that the results discussed below hold for all values of *α*, and hence can be generalized beyond simple binomial partitioning. The overall model coupling cell cycle to expression is illustrated in figure 1 together with a representative trajectory of *x*(*t*).

## 3. Mean protein level for cell-cycle-driven expression

We illustrate an approach based on closing moment dynamics for deriving an exact analytical formula for the mean protein level. The first step is to obtain differential equations describing the time evolution of the statistical moments for *x*(*t*) and *c*_*i*_(*t*). These equations can be derived using the chemical master equation (CME) corresponding to the stochastic model presented in the previous section (see the electronic supplementary material, appendix A). In particular, time evolution of the means (first-order moments) is given by

3.1*a* $$\frac{d\langle c_{1}\rangle }{dt}=\lambda_{n}\langle c_{n}\rangle -\lambda_{1}\langle c_{1}\rangle ,\;\frac{d\langle c_{i}\rangle }{dt}=\lambda_{c-i}\langle c_{i-1}\rangle -\lambda_{i}\langle c_{i}\rangle ,\;i\in \{2,3,\ldots ,n\}$$

and

3.1*b* $$\frac{d\langle x\rangle }{dt}=\left(\sum_{i=1}^{n}k_{i}\langle c_{i}\rangle \right)\langle B\rangle -\frac{\lambda_{n}}{2}\langle xc_{n}\rangle .$$

Steady-state analysis of (3.1a) yields the average value of Bernoulli processes as

3.2 $$\bar{\left\langle c_{i}\right\rangle }=\frac{1/\lambda_{i}}{\sum_{j=1}^{n}(1/\lambda_{j})},$$

which can be interpreted as the fraction of time spent in the cell-cycle stage *C*_*i*_. We use $\bar{\left\langle \;\right\rangle }$ to denote the expected value of a stochastic process as t→∞.

Note that the dynamics of 〈*x*〉 in (3.1b) is ‘not closed’, in the sense that it depends on the second-order moments 〈*xc*_*n*_〉. This leads to the well-known problem of moment closure that often arises in stochastic chemical kinetics [54–61]. It turns out that in this case, the model structure can be exploited to automatically close moment equations. This is done by augmenting the system of equations in (3.1) with the time evolution of moments of the form 〈*xc*_*i*_〉

3.3*a* $$\frac{d\langle xc_{1}\rangle }{dt}=k_{1}\langle B\rangle \langle c_{1}\rangle +\frac{\lambda_{n}}{2}\langle xc_{n}\rangle -\frac{\lambda_{n}}{2}\langle xc_{1}c_{n}\rangle -\lambda_{1}\langle xc_{1}\rangle$$

and

3.3*b* $$\frac{d\langle xc_{i}\rangle }{dt}=k_{i}\langle B\rangle \langle c_{i}\rangle -\lambda_{i}\langle xc_{i}\rangle +\lambda_{i-1}\langle xc_{i-1}\rangle .\;j\in \{2,\ldots ,n\}.$$

At the first look, these equations are unclosed and depend on the third-order moments of the form 〈*xc*_*i*_*c*_*n*_〉. However, exploiting the fact that *c*_*i*_*c*_*j*_=0 from (2.1) leads to trivial closure

3.4 $$\langle xc_{i}c_{n}\rangle =0.$$

After using (3.4) in (3.3a), the mean protein level can be computed exactly by solving a linear dynamical system given by (3.1) and (3.3). At steady state, the linear equations can be solved recursively to yield

3.5 $$\bar{\left\langle xc_{i}\right\rangle }=\frac{\left\langle B\right\rangle }{\lambda_{i}}\frac{\sum_{j=1}^{n}(k_{j}/\lambda_{j})+\sum_{j=1}^{i}(k_{j}/\lambda_{j})}{\sum_{j=1}^{n}(1/\lambda_{j})},$$

where 〈*B*〉 is the mean protein burst size. As *c*_*i*_s are binary random variables, the mean protein level conditioned on the cell-cycle stage (i.e. synchronized cell population) can be obtained as

3.6 $$\bar{\left\langle x\;|\;c_{i}\right\rangle }=\frac{\bar{\left\langle xc_{i}\right\rangle }}{\bar{\left\langle c_{i}\right\rangle }}=\langle B\rangle \left(\sum_{j=1}^{n}\frac{k_{j}}{\lambda_{j}}+\sum_{j=1}^{i}\frac{k_{j}}{\lambda_{j}}\right).$$

Furthermore, using (3.5) and the fact that $\sum_{i=1}^{n}c_{i}=1$,

3.7 $$\bar{\left\langle x\right\rangle }=\sum_{j=1}^{n}\bar{\left\langle xc_{i}\right\rangle }=\frac{\left\langle B\right\rangle }{\sum_{j=1}^{n}(1/\lambda_{j})}\left(\sum_{i=1}^{n}\sum_{j=1}^{n}\frac{k_{j}}{\lambda_{i}\lambda_{j}}+\sum_{i=1}^{n}\sum_{j=1}^{i}\frac{k_{j}}{\lambda_{i}\lambda_{j}}\right).$$

Next, we investigate the mean protein level $\bar{\left\langle x\right\rangle }$ in some limiting cases. Consider equal transition rates between cell-cycle stages λ_*i*_=*n*/*T*, which corresponds to Erlang distributed cell-cycle durations with mean *T* and shape parameter *n*. In this scenario,

3.8 $$\bar{\left\langle x\right\rangle }=\frac{\left\langle B\rangle T(\sum_{i=1}^{n}\sum_{j=1}^{n}k_{j}+\sum_{i=1}^{n}\sum_{j=1}^{i}k_{j}\right)}{n^{2}},$$

and further reduces to

3.9 $$\bar{\left\langle x\right\rangle }=\langle B\rangle Tk\left(\frac{3}{2}+\frac{1}{2n}\right)$$

when the rate of expression bursts *k*_*i*_=*k* is constant throughout the cell cycle. Finally, in the limit of deterministic cell-cycle durations of length *T* (n→∞)

3.10 $$\bar{\left\langle x\right\rangle }=\frac{3\langle B\rangle Tk}{2}.$$

## 4. Protein noise level for cell-cycle-driven expression

The mathematical approach illustrated above is now used to obtain the noise in protein copy numbers. By noise (cell-to-cell variability), we mean the magnitude of fluctuations in *x*(*t*) that can be attributed to two stochastic mechanisms: bursty expression and random partitioning. Note that even in the absence of these mechanisms, there will be cell-cycle-related fluctuations with protein molecules accumulating over time and dividing by half at random cell-division times. To correct for such cell-cycle-driven fluctuations, we define another stochastic process *y*(*t*) that estimates the protein level if expression and partitioning were modelled deterministically. More specifically, within the cell cycle *y*(*t*) evolves according to the following differential equation:

4.1 $$\dot{y}=\langle B\rangle \sum_{i=1}^{n}k_{i}c_{i}(t),$$

which is the deterministic counterpart to the stochastic expression model presented earlier. At the time of cell division, the level is divided exactly by half

4.2 $$y(t)\to \frac{y(t)}{2}$$

with zero partitioning errors, i.e. *α*=0 in (2.5). This allows us to define a new zero-mean stochastic process *z*(*t*) corrected for cell-cycle effects

4.3 z(t):=x(t)−y(t)

that measures the deviation in the protein count in the original stochastic model (*x*) from its expected levels if noise mechanisms were modelled deterministically (*y*). The protein noise level can now be defined through the dimensionless quantity

4.4 $$\eta :=\frac{\bar{\left\langle z^{2}\right\rangle }}{{\bar{\left\langle x\right\rangle }}^{2}},$$

measuring the steady-state variance in *z*(*t*) normalized by the square of the mean level. As $\bar{\left\langle x\right\rangle }=\bar{\left\langle y\right\rangle }$ and $\bar{\left\langle xy\right\rangle }=\bar{\left\langle y^{2}\right\rangle }$ (see the electronic supplementary material, appendix B), it can be rewritten as

4.5 $$\eta =\frac{\bar{\left\langle {\left(x-y\right)}^{2}\right\rangle }}{{\bar{\left\langle x\right\rangle }}^{2}}=\frac{\bar{\left\langle x^{2}\right\rangle }}{{\bar{\left\langle x\right\rangle }}^{2}}-\frac{\bar{\left\langle y^{2}\right\rangle }}{{\bar{\left\langle y\right\rangle }}^{2}}.$$

In the context of prior work, $\bar{\left\langle y^{2}\right\rangle }/{\bar{\left\langle y\right\rangle }}^{2}-1$ is interpreted as the ‘extrinsic noise’ in gene expression resulting from cell-cycle effects. It is typically measured by the covariance in the singe-cell expression of two identical copies of a gene with common cell-cycle regulation [62,63]. By contrast, *η* is the ‘intrinsic noise’ resulting from stochasticity in gene expression and partitioning processes, and is measured by subtracting the extrinsic noise from the total noise $\bar{\left\langle x^{2}\right\rangle }/{\bar{\left\langle x\right\rangle }}^{2}-1$. The results derived here can also be obtained by performing two-colour assay (see the electronic supplementary material, appendix C).

Having appropriately defined the noise level, we next compute it using moment equations. The time evolution of the moments 〈*z*^2^〉 and 〈*z*^2^*c*_*i*_〉 are given by (see the electronic supplementary material, appendix D)

4.6*a* $$\begin{array}{rl} \frac{d\langle z^{2}\rangle }{dt} & =\langle B^{2}\rangle \sum_{i=1}^{n}k_{i}\langle c_{i}\rangle +\frac{\alpha \lambda_{n}}{4}\langle xc_{n}\rangle +\frac{\lambda_{n}}{4}\langle z^{2}c_{1}c_{n}\rangle -\frac{3}{4}\lambda_{n}\langle z^{2}c_{n}\rangle \end{array}$$

4.6*b* $$\begin{array}{rl} \frac{d\langle z^{2}c_{1}\rangle }{dt} & =k_{1}\langle B^{2}\rangle \langle c_{1}\rangle +\frac{\alpha \lambda_{n}}{4}\langle xc_{n}\rangle +\frac{\lambda_{n}}{4}\langle z^{2}c_{n}\rangle -\lambda_{1}\langle z^{2}c_{1}\rangle \end{array}$$

4.6*c* $$\begin{array}{rl} \text{and}\;\frac{d\langle z^{2}c_{i}\rangle }{dt} & =k_{i}\langle B^{2}\rangle \langle c_{i}\rangle -\lambda_{i}\langle z^{2}c_{i}\rangle +\lambda_{i-1}\langle z^{2}c_{i-1}\rangle ,\;i=\{2,\ldots ,i\}, \end{array}$$

and depend on the fourth-order moments 〈*z*^2^*c*_1_*c*_*n*_〉. Exploiting the model structure as before, it follows from (2.1) that 〈*z*^2^*c*_1_*c*_*n*_=0〉, and (3.1), (3.3) and (4.6) constitute a ‘closed’ set of linear differential equations. Steady-state analysis yields the following noise level (see the electronic supplementary material, appendix D)

4.7 $$\eta =\underset{\mathrm{bursty}\;\mathrm{synthesis}}{\underbrace{\left(\frac{1}{3}+\frac{2}{3}\frac{1}{1+\beta }\right)\frac{\left\langle B^{2}\right\rangle }{\left\langle B\right\rangle }\frac{1}{\bar{\left\langle x\right\rangle }}}}+\underset{\mathrm{partitioning}\;\mathrm{errors}}{\underbrace{\frac{2\alpha }{3}\frac{\beta }{1+\beta }\frac{1}{\bar{\left\langle x\right\rangle }}}}$$

that is inversely proportional to the mean $\bar{\left\langle x\right\rangle }$. The noise can be decomposed into two terms: the first term represents the contribution from protein synthesis in random bursts and depends on the statistical moments of the protein burst size *B*. The second term is the contribution from partitioning errors and depends linearly on *α*. Recall that *α* measures the degree of randomness in partitioning of molecules between daughter cells and is defined through (2.5). Interestingly, results show that the effect of cell-cycle regulation on the noise level can be quantified through a single dimensionless parameter

4.8 $$\beta =\frac{\sum_{i=1}^{n}\sum_{j=1}^{n}(k_{j}/\lambda_{i}\lambda_{j})}{\sum_{i=1}^{n}\sum_{j=1}^{i}(k_{j}/\lambda_{i}\lambda_{j})}$$

that is uniquely determined by the number of cell-cycle stages in the model (*n*), transition rates between stages (λ_*i*_), and protein synthesis rates across stages (*k*_*i*_). Note from (4.7) that *β* affects the noise terms in opposite ways—any coupling of cell cycle to expression that increases *β* will attenuate the contribution from bursty expression but amplifies the contribution from partitioning errors. Finally, we point out that in the case of non-bursty expression (*B*=1 with probability one) and binomial partitioning (*α*=1)

4.9 $$\eta =\frac{1}{\bar{\left\langle x\right\rangle }}$$

and the noise level is always consistent with that of a Poisson distribution^1^ irrespective of the value of *β*, and hence the form of cell-cycle regulation.

## 5. Optimal cell-cycle regulation to minimize noise

We explore how different forms of cell-cycle regulation affect *η* and begin with the simplest case of a constant synthesis rate *k*_*i*_=*k*, *i*∈{1,2,…,*n*} throughout the cell cycle. This case would correspond to a scenario where the net rate of expression (across all copies of a gene) remains invariant to replication-associated changes in gene dosage, as has recently been shown in different organisms [34,33]. Further assuming equal transition rates λ_*i*_=*n*/*T* (Erlang distributed cell-cycle durations)

5.1 $$\beta =\frac{2n}{n+1}\;,$$

which reduces to *β*=2 as n→∞. Thus, in this important limit of no cell-cycle regulation (equal *k*_*i*_s) and deterministic cell-cycle duration (large *n*),

5.2 $$\eta =\frac{5}{9}\frac{\left\langle B^{2}\right\rangle }{\left\langle B\right\rangle }\frac{1}{\bar{\left\langle x\right\rangle }}+\frac{4\alpha }{9}\frac{1}{\bar{\left\langle x\right\rangle }}\;\mathrm{for}\;\beta =2.$$

Next, consider the following strategies for coupling cell cycle to gene expression:

(i) The burst arrival rate is assumed to increase by two-fold at the cell-cycle midpoint due to gene duplication. Assuming even *n*, this corresponds to
  5.3*a* $$k_{i}=k,\;i\in \left\{1,\ldots ,\frac{n}{2}\right\}$$
  and
  5.3*b* $$k_{i}=2k,\;i\in \left\{\frac{n}{2}+1,\ldots ,n\right\}.$$
(ii) Expression only occurs at the start of cell cycle, i.e. *k*_1_=*k* and all other *k*_*i*_s are zero.
(iii) Expression only occurs at the end of cell cycle, i.e. *k*_*n*_=*k* and all other *k*_*i*_s are zero.
(iv) Expression only occurs at the cell cycle midpoint, i.e. *k*_*n*/2_=*k* and all other *k*_*i*_s are zero.

**Figure 2.**
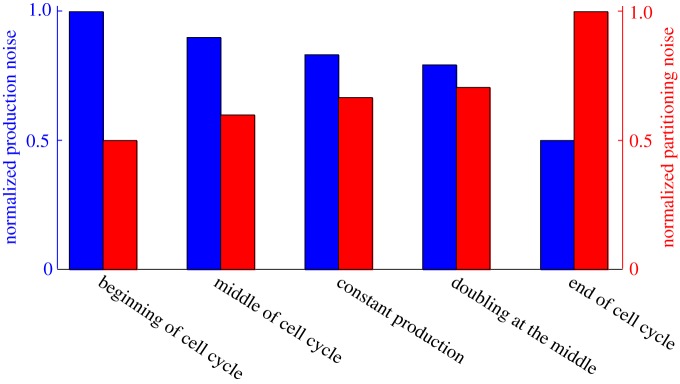
Noise comparison for different strategies coupling cell cycle to gene expression. The noise from bursty expression (left) and partitioning errors (right) as given by (4.7) are shown for five different strategies: expression only at the start of the cell cycle, expression only at the cell-cycle midpoint, constant mRNA synthesis rate throughout the cell cycle, doubling of synthesis rate at the cell-cycle midpoint and expression only towards the end of the cell cycle. While noise from bursty expression is minimized in the latter strategy, contribution from partitioning errors are lowest if expression occurs only at the beginning of the cell cycle. The cell cycle was modelled by choosing *n*=20 stages with equal transition rates, i.e. stages have equal mean duration. The duration of each stage is an exponentially distributed random variable. The production rates *k*_*i*_ were chosen so as to have the same mean protein level per cell across all cases.

For a mathematically controlled comparison, the parameter *k* is adjusted using (3.7) from case-to-case so as to maintain a fixed average number of protein molecules. It is important to point out that strategies ii–iv above correspond to expression only occurring at specific instants in the cell-cycle, with expression turned off for the remainder of the cycle. Our results show that the noise contribution from bursty expression is different depending on when the proteins are synthesized, and it is the highest (lowest) when expression occurs at the start (end) of the cell cycle (figure 2). Furthermore, as expected from (4.7), the noise contribution from partitioning errors exhibits a completely opposite trend.

**Figure 3.**
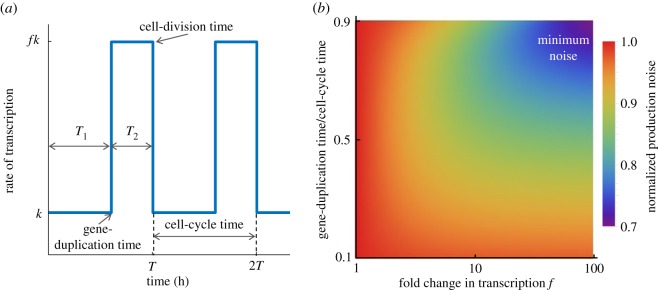
Protein noise level from bursty expression is minimal when gene-duplication event is at the cell-cycle end, and fold-change in transcription is high. (*a*) The rate of transcription within the cell cycle is modelled as a step function—it is equal to *k* (*fk*) before (after) the gene-duplication event, where *f* is the fold-change in transcription. The event is assumed to occur at time *T*_1_ since the start of the cell cycle, and cell division occurs at time *T*=*T*_1_+*T*_2_. After division, the rate again resets to *k*. The times *T*_1_ and *T*_2_ are assumed to be deterministic. (*b*) The noise contribution from bursty expression is plotted as a function of *T*_1_/*T* and *f*. The value of *k* is changed so as to keep the mean protein level fixed. Noise levels are normalized to the noise when protein is expressed at a constant rate throughout the cell cycle (*f*=1). The plot reveals that the noise in smallest when *T*_1_/*T* is close to 1, and *f* is large.

Interestingly, a twofold increase in the protein expression rate (due to gene duplication) at the cell-cycle midpoint leads to a lower noise contribution from bursty synthesis, when compared with a constant rate throughout the cell cycle (figure 2). Is it possible to further reduce noise levels by changing the timing of genome-duplication? This question is particularly relevant since genes can be duplicated at different times in the cell cycle, and depending on dosage-compensation mechanisms, have different fold-changes in transcription rates upon duplication [32]. To investigate this scenario, we consider an *f*-fold change in the synthesis rate (from *k* to *fk*) that occurs at some time *T*_1_ from the start of the cell cycle. Noise is investigated as a function of *T*_1_ and *f*, while keeping a fixed average protein level through alterations in *k* (figure 3). Intriguingly, our analysis reveals that for a fixed *T*_1_, noise contribution from bursty synthesis always decreases with increasing *f* (figure 3). Moreover, the minimal noise is obtained when *f* is as large as possible, and the duplication event occurs close to the cell-cycle end, i.e. the protein is expressed at a small basal rate within the cell cycle, and the rate is increased for a small time window just before division (figure 3).

The above results motivate a related but more general question: is there an optimal way to express a protein during the cell cycle that maximizes/minimizes protein noise levels? As the form of cell-cycle regulation impacts *η* through *β*, this amounts to choosing *k*_*i*_s so as to maximize/minimize it. Our result show that *β* is bounded from both below and above (see the electronic supplementary material, appendix E)

5.4 $$1\leq \beta \leq \beta_{\max}=\frac{1/\lambda_{1}+1/\lambda_{2}+\cdots +1/\lambda_{n}}{1/\lambda_{n}}.$$

The minimal value of *β*=1 is attained when expression only occurs at the start of the cell cycle, i.e. a non-zero *k*_1_, and all other *k*_*i*_s are zero. In this case,

5.5 $$\eta =\frac{2}{3}\frac{\left\langle B^{2}\right\rangle }{\left\langle B\right\rangle }\frac{1}{\bar{\left\langle x\right\rangle }}+\frac{\alpha }{3}\frac{1}{\bar{\left\langle x\right\rangle }}\;\mathrm{for}\;\beta =1,$$

with the lowest noise contribution from partitioning errors but the highest contribution from bursty synthesis. By contrast, the maximum value of $\beta =\beta_{\max}$ is attained when expression only occurs at the end of the cell cycle, i.e. a non-zero *k*_*n*_, and all other *k*_*i*_s are relatively small or zero. Note from (5.4) that $\beta_{\max}\to \infty$ as $\lambda_{n}\to \infty$ (time spent in stage *C*_*n*_ approaches zero), in which case

5.6 $$\eta =\frac{1}{3}\frac{\left\langle B^{2}\right\rangle }{\left\langle B\right\rangle }\frac{1}{\bar{\left\langle x\right\rangle }}+\frac{2\alpha }{3}\frac{1}{\bar{\left\langle x\right\rangle }}\;\mathrm{for}\;\beta =\infty ,$$

and the noise contribution from bursty synthesis is minimal.

**Figure 4.**
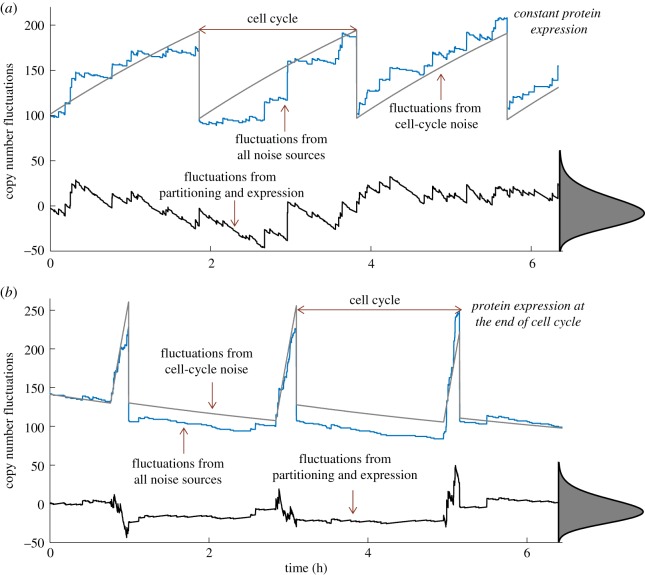
Synthesis of proteins towards the end of the cell cycle minimizes fluctuations in copy numbers. Protein level in an individual cell across multiple cell cycles for two strategies: a fixed transcription rate throughout the cell cycle (*a*) and transcription increases drastically just before cell division (*b*). In both cases, we assumed that there exists a small degradation of protein through the cell cycle. Trajectories obtained via Monte Carlo (MC) simulations are shown for the stochastic model (blue) and a reduced model where noise mechanisms are modelled deterministically (grey). These levels are subtracted to obtain a zero-mean stochastic process *z*(*t*), where fluctuations resulting from the cell cycle are removed (black). Steady-state distribution of *z* obtained from 20 000 MC simulation runs is shown on the right, and the bottom strategy leads to lower variability in *z* for the same mean protein level. Cell cycle and expression were modelled as in figure 1 and burst arrival rates were chosen so as to ensure an average protein copy number of 150 molecules per cell in both cases.

In summary, consistent with the finding of figure 3, if bursty expression is the dominant source of noise (high *B* and low *α*), then *η* is minimized for a given $\bar{\left\langle x\right\rangle }$ when the protein is made in the shortest time window just before cell division (figure 4). On the other hand, if randomness in partitioning error is dominant (low *B* and high *α*), the optimal strategy is to make the protein just after cell division. Furthermore, these optimal strategies also minimize stochastic variation in protein counts among synchronized cells, where all cells are in the same cell-cycle stage (see the electronic supplementary material, appendix F).

## 6. Conclusion

Theoretical models of stochastic gene expression have played a pivotal role in understanding how noise mechanisms and biologically relevant parameters generate differences in protein/mRNA population counts between isogenic cells [64–69]. Here, we have expanded this theory to consider cell-cycle-regulated genes. Our approach involves a general model of the cell cycle, with a cell transitioning through an arbitrary number of stages from birth to division. The protein is assumed to be expressed in random bursts, and the rate at which bursts arrive varies arbitrarily with cell-cycle stage. In the case of translational bursting of proteins from mRNA, the burst arrive rate corresponds to the mRNA synthesis (transcription) rate. By contrast, for transcriptional bursting of mRNAs, the burst arrive rate corresponds to the frequency with which a promoter become transcriptionally active. The key contribution of this work is derivations of (3.7) and (4.7) that predict the protein mean and noise levels for a given form of cell-cycle regulation.

Derivation of noise formulae enables uncovering of optimal cell-cycle regulation strategies to minimize *η* for a fixed mean protein level. In the physiological case of large bursts (*B*≫1) and binomial partitioning of proteins between daughter cells (*α*=1), the contribution from bursty synthesis dominates *η*. Our results show that in this scenario, expression of the protein just before division is the optimal strategy (figure 3). Intuitively, such a strategy can be understood in the context of the number of burst events from birth to division needed to maintain a given $\bar{\left\langle x\right\rangle }$ throughout the cell cycle. It turns out that this number is highly dependent on the form of cell-cycle regulation. Hence, any strategy that requires more burst events to maintain the same mean protein level lowers noise through more effective averaging of the underlying bursty process, albeit being more energy inefficient. For example, if protein production only occurs at the end of the cell cycle, then on average $\bar{\left\langle x\right\rangle }$ number of proteins have to be added just before cell division. This corresponds to $\bar{\left\langle x\right\rangle }/\langle B\rangle$ number of burst events per cell cycle. If proteins were only expressed at the start of the cell cycle, then one needs to add only $\bar{\left\langle x\right\rangle }/2$ number of molecules, half as much as the earlier strategy. If proteins were made at a constant synthesis rate throughout the cell cycle, then on average $2\bar{\left\langle x\right\rangle }/3$ number of molecules are added per cell cycle, which is higher than the early-expression strategy but lower than the late-expression strategy. In summary, gene product synthesis just before division requires production of the most number of protein molecules to maintain a fixed mean level within the cell cycle, and hence provides the most effective noise buffering through averaging of burst events. Next, we provide two recent examples of proteins that are indeed expressed in this manner.

The green alga *Chlamydomonas reinhardtii* has a prolonged *G*_1_ phase, where the size of a newborn cell increases by more than twofold. This long *G*_1_ phase is followed by an *S*/*M* phase. Here, the cell undergoes multiple DNA replication and fission cycles creating 2^*d*^ daughter cells, where *d* is the number of rounds of division. Recent studies suggest that the number of rounds of division is controlled by a protein CDKG1, that is only expressed just before exit from *G*_1_ [70]. Another example is the protein Whi5 in budding yeast *S. cerevisiae* the level of which controls the transition of cells past the Start checkpoint. This protein is not expressed in *G*_1_ and is only synthesized late in the cell cycle [71,72]. While such selective expression of these proteins plays a critical role in coupling cell size to cell-cycle decision, it may also minimize intrinsic fluctuations in protein levels from the innate stochasticity in gene expression. Clearly, a more systematic study exploring the role of noisy expression on the fidelity of these the cell cycle decisions is warranted.

It is important to point out that our analysis made various simplifying assumptions. For example, protein synthesis was assumed to occur in instantaneous translational and transcriptional bursts that correspond to short-lived mRNAs and unstable active promoter states, respectively [73]. Furthermore, cell-cycle durations were independent random variables, implying no correlation between the division times of mother and daughter cells. In the electronic supplementary material, appendix G, we have considered a detailed expanded model that explicitly includes promoter switching, mRNA dynamics and fluctuations in cell-cycle durations with memory across generations. Model analysis using Monte Carlo simulations reveals that consistent with insights from simplified models, expression at the end of the cell cycle provides the best noise buffering in protein levels from stochastic expression.

While our analysis is restricted to non-regulated genes, future work will consider feedback regulation in expression. Feedback can be incorporated by allowing the burst frequency to be a linear function of the protein level (i.e. linear feedback), or by considering rates based on nonlinear Hill functions that often result in bistable and oscillatory gene circuits [74,75]. Another key point is that in our study, expression is cell-cycle dependent, but cell-size independent. Thus, another direction of work is to include time evolution of cell volume, and consider size-dependent expression. This will allow investigation of both concentration and copy number of gene products in single cells, and some recent work on modelling stochastic dynamics of cell size has already been done [76–79].

## Supplementary Material

Supplementary Information with additional proofs and mathematical details

## Footnotes

1 The coefficient of variation squared for a Poisson distributed random variable is inverse of its mean.

## Data accessibility

Codes used to build figures are available at Dryad Data Repository: http://dx.doi.org/10.5061/dryad.7kv8r [80].
