# Supplementary material for: Effects of cell-cycle-dependent expression on random fluctuations in protein levels
Source: R Soc Open Sci. 2016 Dec 7;3(12):160578. doi: 10.1098/rsos.160578 (PMC5210684; doi:10.1098/rsos.160578)
Supplement: Supplementary Information with additional proofs and mathematical details [file rsos160578supp1.pdf]

Effects of cell-cycle dependent expression on random fluctuations in protein levels

Contents

A Moment dynamics of x **2**

B Moment dynamics of y **3**

C Intrinsic noise obtained from two-color assay **6**

D Moments dynamics of z **8**

E Optimal value of β **11**

F Cell-to-cell variability in synchronized cells **12**

G Incorporating cell-cycle times correlations, promoter switching, and mRNA dynamics in the model **14**

Appendix A

Moment dynamics of x

Based on standard stochastic formulation of chemical kinetics [1, 2], the model describing x contains the following stochastic events

$$\text{Protein production:} \qquad x \xmapsto{k_i c_i p_j} x + j, \tag{A.1a}$$

$$\text{Cell stage evolution:} \qquad c_i \xmapsto{\lambda_i c_i} c_i - 1, \quad c_{i+1} \xmapsto{\lambda_i c_i} c_{i+1} + 1, \tag{A.1b}$$

$$\text{Cell division:} \qquad x \xmapsto{\lambda_n c_n} x_+, \quad c_n \xmapsto{\lambda_n c_n} c_n - 1, \quad c_1 \xmapsto{\lambda_n c_n} c_1 + 1, \tag{A.1c}$$

where the probability of having a burst of j molecules is given by p_j. Whenever an event occurs, the states of the system change based on the stochiometries given in (A.1). On top of the arrows we showed the event propensity function $\psi(x, c) = \psi(x, c_1, c_2, \ldots, c_n)$, which determines how often reactions occur, i.e., the probability that an event occurs in the next infinitesimal time interval $(t, t + dt]$ is $\psi(x, c)dt$. Time derivative of the expected value of any function $\varphi(x, c) = \varphi(x, c_1, c_2, \ldots, c_n)$ for this system can be written as [3]

$$\frac{d\langle\varphi(x, c)\rangle}{dt} = \left\langle \sum_{Events} \Delta\varphi(x, c) \times \psi(x, c) \right\rangle, \tag{A.2}$$

where $\Delta\varphi(x, c) = \Delta\varphi(x, c_1, c_2, \ldots, c_n)$ is the change in $\varphi(x, c)$ when an event occurs. Choosing φ to be x and $c_i, i = \{1, 2, \ldots, n\}$ results in the equation (3.1) in the main article.

Appendix B

Moment dynamics of y

The model describing x and y includes the stochastic events

$$\text{Protein production:} \quad x \xmapsto{k_i c_i p_j} x + j, \tag{B.1a}$$

$$\text{Cell stage evolution:} \quad c_i \xmapsto{\lambda_i c_i} c_i - 1, \; c_{i+1} \xmapsto{\lambda_i c_i} c_{i+1} + 1, \tag{B.1b}$$

$$\text{Cell division:} \quad x \xmapsto{\lambda_n c_n} x_+, \; y \xmapsto{\lambda_n c_n} y_+, \; c_n \xmapsto{\lambda_n c_n} c_n - 1, \; c_1 \xmapsto{\lambda_n c_n} c_1 + 1, \tag{B.1c}$$

and the deterministic production of y

$$\dot{y} = \left(\sum_{i=1}^{n} k_i c_i \right) \langle B \rangle. \tag{B.2}$$

Time derivative of the expected value of any function $\varphi(x, y, c) = \varphi(x, y, c_1, c_2, \ldots, c_n)$ for this system can be written as [3]

$$\frac{d\langle \varphi(x, y, c) \rangle}{dt} = \left\langle \sum_{Events} \Delta\varphi(x, y, c) \times \psi(x, y, c) \right\rangle + \left\langle \left(\sum_{i=1}^{n} k_i c_i \right) \frac{\partial \varphi(x, y, c)}{\partial z} \langle B \rangle \right\rangle, \tag{B.3}$$

where the first term in the right-hand side is contributed from stochastic events and the second one is contributed from (B.2). The propensity function of the events is given by $\psi(x, y, c) = \psi(x, y, c_1, c_2, \ldots, c_n)$, and $\Delta\varphi(x, y, c) = \Delta\varphi(x, y, c_1, c_2, \ldots, c_n)$ is the change in $\varphi(x, y, c)$ when an event occurs. The mean dynamics of y can be written by choosing φ to be y

$$\frac{d\langle y \rangle}{dt} = \left(\sum_{i=1}^{n} k_i \langle c_i \rangle \right) \langle B \rangle - \frac{\lambda_n}{2} \langle y c_n \rangle. \tag{B.4}$$

Dynamics of $\langle y\rangle$ is not closed and depends to moments $\langle yc_n\rangle$, hence in order to have a closed set of equations we add new moments dynamics by selecting φ to be yc_i

$$\frac{d\langle yc_1\rangle}{dt} = k_1\langle B\rangle\langle c_1\rangle + \frac{\lambda_n}{2}\langle yc_n\rangle - \lambda_1\langle yc_1\rangle, \tag{B.5a}$$
$$\frac{d\langle yc_i\rangle}{dt} = k_i\langle B\rangle\langle c_i\rangle - \lambda_i\langle yc_i\rangle + \lambda_{i-1}\langle yc_{i-1}\rangle, \quad j \in \{2,\dots,n\}. \tag{B.5b}$$

Dynamics of $\langle y\rangle$ and $\langle yc_i\rangle,\ j \in \{1,\dots,n\}$ are the same as dynamics of $\langle x\rangle$ and $\langle xc_i\rangle,\ j \in \{1,\dots,n\}$ presented in (3.1b) and (3.3) in the main text, hence $\overline{\langle x\rangle} = \overline{\langle y\rangle}$ and $\overline{\langle xc_i\rangle} = \overline{\langle yc_i\rangle}$.

Further, dynamics of $\langle xy\rangle$ can be written as

$$\frac{d\langle xy\rangle}{dt} = \left(\sum_{i=1}^{n} k_i\left(\langle xc_i\rangle + \langle yc_i\rangle\right)\right)\langle B\rangle - \frac{\lambda_n}{4}\langle xyc_n\rangle. \tag{B.6}$$

In order to have a closed set of equations we add dynamics of $\langle xyc_i\rangle$

$$\frac{d\langle xyc_1\rangle}{dt} = k_1\left(\langle xc_1\rangle + \langle yc_1\rangle\right)\langle B\rangle + \frac{\lambda_n}{4}\langle xyc_n\rangle - \lambda_1\langle xyc_1\rangle, \tag{B.7a}$$
$$\frac{d\langle xyc_i\rangle}{dt} = k_i\left(\langle xc_i\rangle + \langle yc_i\rangle\right)\langle B\rangle - \lambda_i\langle xyc_i\rangle + \lambda_{i-1}\langle xyc_{i-1}\rangle, \ i = \{2,\dots,i\}. \tag{B.7b}$$

By having a closed set of equations related to xy, in the next step we add dynamics of $\langle y^2\rangle$ and $\langle y^2c_i\rangle$

$$\frac{d\langle y^2\rangle}{dt} = 2\left(\sum_{i=1}^{n} k_i\langle yc_i\rangle\right)\langle B\rangle - \frac{\lambda_n}{4}\langle y^2c_n\rangle, \tag{B.8a}$$
$$\frac{d\langle y^2c_1\rangle}{dt} = 2k_1\langle yc_1\rangle\langle B\rangle + \frac{\lambda_n}{4}\left\langle y^2c_n\right\rangle - \lambda_1\langle y^2c_1\rangle, \tag{B.8b}$$
$$\frac{d\langle y^2c_i\rangle}{dt} = 2k_i\langle yc_i\rangle\langle B\rangle - \lambda_i\langle y^2c_i\rangle + \lambda_{i-1}\langle y^2c_{i-1}\rangle, \ i = \{2,\dots,i\}. \tag{B.8c}$$

Using the fact that $\overline{\langle x\rangle} = \overline{\langle y\rangle}$ and $\overline{\langle xc_i\rangle} = \overline{\langle yc_i\rangle}$, equations (B.6), (B.7), and (B.8) in steady-state results in $\overline{\langle y^2\rangle} = \overline{\langle xy\rangle}$ and $\overline{\langle y^2 c_i\rangle} = \overline{\langle xyc_i\rangle}$.

Appendix C

Intrinsic noise obtained from two-color assay

In this section we show that the results obtained here can be derived from two-color assay. Consider two identical proteins x_1 and x_2 which their dynamics are exactly the same as protein x in the main article. The model describing x_1 and x_2 includes the stochastic events

$$\text{Protein } x_1 \text{ production:} \quad x_1 \xmapsto{k_i c_i p_{j_1}} x_1 + j_1, \tag{C.1a}$$

$$\text{Protein } x_2 \text{ production:} \quad x_2 \xmapsto{k_i c_i p_{j_2}} x_2 + j_2, \tag{C.1b}$$

$$\text{Cell stage evolution:} \quad c_i \xmapsto{\lambda_i c_i} c_i - 1, \ c_{i+1} \xmapsto{\lambda_i c_i} c_{i+1} + 1, \tag{C.1c}$$

$$\text{Cell division:} \quad x_1 \xmapsto{\lambda_n c_n} x_{1+}, \ x_2 \xmapsto{\lambda_n c_n} x_{2+}, \ c_n \xmapsto{\lambda_n c_n} c_n - 1, \ c_1 \xmapsto{\lambda_n c_n} c_1 + 1. \tag{C.1d}$$

Note that the mean burst sizes of x_1 and x_2 are equal to the mean burst size of x. For this model the intrinsic noise can be quantified as

$$\eta = \frac{\overline{\langle x_1^2 \rangle} - \overline{\langle x_1 x_2 \rangle}}{\overline{\langle x_1 \rangle}^2} \tag{C.2}$$

[4].

Since dynamics of x_1 and x_2 are exactly the same as x, we have $\overline{\langle x_1 \rangle} = \overline{\langle x_2 \rangle} = \overline{\langle x \rangle}$ and $\overline{\langle x_1^2 \rangle} = \overline{\langle x_2^2 \rangle} = \overline{\langle x^2 \rangle}$. Further in Appendix B we show that $\overline{\langle x c_i \rangle} = \overline{\langle y c_i \rangle}$ hence $\overline{\langle x_1 c_i \rangle} = \overline{\langle x_2 c_i \rangle} = \overline{\langle y c_i \rangle}$. In the next we show that $\overline{\langle x_1 x_2 \rangle} = \overline{\langle y^2 \rangle}$. Time derivative of the expected value of any function $\varphi(x_1, x_2, c) = \varphi(x_1, x_2, c_1, c_2, \ldots, c_n)$ for this system can

be written as [3]

$$\frac{d\langle\varphi(x_1,x_2,c)\rangle}{dt} = \left\langle \sum_{Events} \Delta\varphi(x_1,x_2,c) \times \psi(x_1,x_2,c) \right\rangle, \tag{C.3}$$

where the propensity function of the events is given by $\psi(x_1,x_2,c) = \psi(x_1,x_2,c_1,c_2,\ldots,c_n)$, and $\Delta\varphi(x_1,x_2,c) = \Delta\varphi(x_1,x_2,c_1,c_2,\ldots,c_n)$ is the change in $\varphi(x_1,x_2,c)$ when an event occurs. The mean dynamics of x_1x_2 can be written by choosing φ to be x_1x_2

$$\frac{d\langle x_1x_2\rangle}{dt} = \left(\sum_{i=1}^{n} k_i\langle x_1c_i\rangle\right)\langle B\rangle + \left(\sum_{i=1}^{n} k_i\langle x_2c_i\rangle\right)\langle B\rangle - \frac{\lambda_n}{4}\langle x_1x_2c_n\rangle. \tag{C.4a}$$

In order to have a closed set of equations, we add new moments dynamics by selecting φ to be $x_1x_2c_i$

$$\frac{d\langle x_1x_2c_1\rangle}{dt} = k_1\langle x_1c_1\rangle\langle B\rangle + k_1\langle x_2c_1\rangle\langle B\rangle + \frac{\lambda_n}{4}\langle x_1x_2c_n\rangle - \lambda_1\langle x_1x_2c_1\rangle, \tag{C.5a}$$

$$\frac{d\langle x_1x_2c_i\rangle}{dt} = k_i\langle x_1c_i\rangle\langle B\rangle + k_i\langle x_2c_i\rangle\langle B\rangle - \lambda_i\langle x_1x_2c_i\rangle + \lambda_{i-1}\langle x_1x_2c_{i-1}\rangle,\ i = \{2,\ldots,i\}. \tag{C.5b}$$

Using the fact that $\overline{\langle x_1c_i\rangle} = \overline{\langle x_1c_i\rangle} = \overline{\langle yc_i\rangle}$, equations (C.4) and (C.5) in steady-state are exactly the same as (B.8) in steady-state. Hence $\overline{\langle y^2\rangle} = \overline{\langle x_1x_2\rangle}$ and $\overline{\langle y^2c_i\rangle} = \overline{\langle x_1x_2c_i\rangle}$. It results in

$$\eta = \frac{\overline{\langle x^2\rangle}}{\overline{\langle x\rangle}^2} - \frac{\overline{\langle y^2\rangle}}{\overline{\langle y\rangle}^2} = \frac{\overline{\langle x_1^2\rangle} - \overline{\langle x_1x_2\rangle}}{\overline{\langle x_1\rangle}^2}. \tag{C.6}$$

Appendix D

Moments dynamics of z

The random variable z is governed via

$$z(t) \mapsto z(t) + B, \tag{D.1a}$$

$$\dot{z} = -\left(\sum_{i=1}^{n} k_i c_i\right) \langle B \rangle. \tag{D.1b}$$

Further in the time of division, z_+ is defined as

$$\langle z_+(t_s) | z(t_s) \rangle = \frac{z(t_s)}{2}, \quad \left\langle z_+^2(t_s) - \langle z_+(t_s) \rangle^2 \middle| z(t_s) \right\rangle = \frac{\alpha x(t_s)}{4}. \tag{D.2}$$

Hence the model by taking into account z contains the following stochastic events

$$\text{Protein production:} \quad x \xmapsto{k_i c_i p_j} x + j, \; z \xmapsto{k_i c_i p_j} z + j, \tag{D.3a}$$

$$\text{Cell stage evolution:} \quad c_i \xmapsto{\lambda_i c_i} c_i - 1, \; c_{i+1} \xmapsto{\lambda_i c_i} c_{i+1} + 1, \tag{D.3b}$$

$$\text{Cell division:} \quad x \xmapsto{\lambda_n c_n} x_+, \; z \xmapsto{\lambda_n c_n} z_+, \; c_n \xmapsto{\lambda_n c_n} c_n - 1, \; c_1 \xmapsto{\lambda_n c_n} c_1 + 1, \tag{D.3c}$$

and deterministic dynamics of z given in (D.1b). Time derivative of the expected value of any function $\varphi(x, z, c) = \varphi(x, z, c_1, c_2, \ldots, c_n)$ for this system can be written as [3]

$$\frac{d\langle \varphi(x, z, c) \rangle}{dt} = \left\langle \sum_{Events} \Delta\varphi(x, z, c) \times \psi(x, z, c) \right\rangle - \left\langle \left(\sum_{i=1}^{n} k_i c_i\right) \frac{\partial \varphi(x, z, c)}{\partial z} \langle B \rangle \right\rangle, \tag{D.4}$$

where the first term in the right-hand side is contributed from stochastic events and the

second one is contributed from (D.1b). The propensity function of the events is given by $\psi(x,z,c) = \psi(x,z,c_1,c_2,\ldots,c_n)$, and $\Delta\varphi(x,z,c) = \Delta\varphi(x,z,c_1,c_2,\ldots,c_n)$ is the change in $\varphi(x,z,c)$ when an event occurs.

By choosing φ to be z^2 and $z^2c_i, i=\{1,\ldots,i\}$ we have the following moment dynamics

$$\frac{d\langle z^2\rangle}{dt} = \left(\sum_{i=1}^{n} k_i\langle c_i\rangle\right)\langle B^2\rangle + \frac{1}{4}\alpha\lambda_n\langle xc_n\rangle - \frac{3}{4}\lambda_n\langle z^2c_n\rangle, \tag{D.5a}$$

$$\frac{d\langle z^2c_1\rangle}{dt} = k_1\langle B^2\rangle\langle c_1\rangle + \frac{1}{4}\alpha\lambda_n\left\langle xc_n\right\rangle + \frac{\lambda_n}{4}\left\langle z^2c_n\right\rangle - \lambda_1\langle z^2c_1\rangle, \tag{D.5b}$$

$$\frac{d\langle z^2c_i\rangle}{dt} = k_i\langle B^2\rangle\langle c_i\rangle - \lambda_i\langle z^2c_i\rangle + \lambda_{i-1}\langle z^2c_{(i-1)}\rangle,\ i=\{2,\ldots,i\}\,. \tag{D.5c}$$

Note that just one of the binary states c_i can be 1 at a time, thus $\overline{\langle z^2\rangle} = \sum_{i=1}^{n}\overline{\langle z^2c_i\rangle}$. In order to calculate the terms $\overline{\langle z^2c_i\rangle}$ we need to express the term $\overline{\langle z^2c_n\rangle}$ as the first step. This term can be calculated by analyzing equation (D.5a) in steady-state

$$\overline{\langle z^2c_n\rangle} = \frac{4}{3\lambda_n}\frac{\sum_{j=1}^{n}\frac{k_j}{\lambda_j}}{\sum_{j=1}^{n}\frac{1}{\lambda_j}}\langle B^2\rangle + \frac{2\alpha}{3\lambda_n}\frac{\sum_{j=1}^{n}\frac{k_j}{\lambda_j}}{\sum_{j=1}^{n}\frac{1}{\lambda_j}}\langle B\rangle. \tag{D.6}$$

By using a recursive process we calculate moments $\overline{\langle z^2c_i\rangle}$: we calculate $\overline{\langle z^2c_1\rangle}$ by substituting equation (D.6) in equation (D.5b). Then we use the definition of $\overline{\langle z^2c_1\rangle}$ to calculate $\overline{\langle z^2c_2\rangle}$ from equation (D.5c) and so on

$$\overline{\langle z^2c_i\rangle} = \frac{1}{3\lambda_i}\frac{\sum_{j=1}^{n}\frac{k_j}{\lambda_j}}{\sum_{j=1}^{n}\lambda_j}\langle B^2\rangle + \frac{1}{\lambda_i}\frac{\sum_{j=1}^{i}\frac{k_j}{\lambda_j}}{\sum_{j=1}^{n}\frac{1}{\lambda_j}}\langle B^2\rangle + \frac{2\alpha}{3\lambda_i}\frac{\sum_{j=1}^{n}\frac{k_j}{\lambda_j}}{\sum_{j=1}^{n}\frac{1}{\lambda_j}}\langle B\rangle. \tag{D.7}$$

Summing up all the term in equation (D.7) results in $\overline{\langle z^2\rangle}$

$$\overline{\langle z^2\rangle} = \frac{1}{3\sum_{j=1}^{n}\frac{1}{\lambda_j}}\sum_{i=1}^{n}\sum_{j=1}^{n}\frac{k_j}{\lambda_i\lambda_j}\langle B^2\rangle + \frac{1}{\sum_{j=1}^{n}\frac{1}{\lambda_j}}\sum_{i=1}^{n}\sum_{j=1}^{i}\frac{k_j}{\lambda_i\lambda_j}\langle B^2\rangle + \frac{4\alpha}{3\sum_{j=1}^{n}\frac{1}{\lambda_j}}\sum_{i=1}^{n}\sum_{j=1}^{n}\frac{k_j}{\lambda_i\lambda_j}\langle B\rangle. \tag{D.8}$$

Finally, protein fluctuatios level can be written as

$$\eta = \frac{\overline{\langle z^2 \rangle}}{\overline{\langle x \rangle}^2} = \left(\frac{1}{3} + \frac{2}{3} \frac{1}{1+\beta} \right) \frac{\langle B^2 \rangle}{\langle B \rangle} \frac{1}{\overline{\langle x \rangle}} + \frac{2\alpha}{3} \frac{\beta}{1+\beta} \frac{1}{\overline{\langle x \rangle}}, \tag{D.9}$$

where

$$\beta = \frac{\sum_{i=1}^{n} \sum_{j=1}^{n} \frac{k_j}{\lambda_i \lambda_j}}{\sum_{i=1}^{n} \sum_{j=1}^{i} \frac{k_j}{\lambda_i \lambda_j}}. \tag{D.10}$$

Appendix E

Optimal value of β

From (D.9) it is clear that minimum production noise occurs when β is maximum, and minimum value of partitioning noise happens when β is minimum. β can be written as

$$\beta = \frac{\sum_{i=1}^{n}\sum_{j=1}^{n}\frac{k_j}{\lambda_i\lambda_j}}{\sum_{i=1}^{n}\sum_{j=1}^{i}\frac{k_j}{\lambda_i\lambda_j}} = \frac{\frac{k_1}{\lambda_1}a_1 + \frac{k_2}{\lambda_2}a_1 + \ldots + \frac{k_n}{\lambda_n}a_1}{\frac{k_1}{\lambda_1}a_1 + \frac{k_2}{\lambda_2}a_2 + \ldots + \frac{k_n}{\lambda_n}a_n}, \tag{E.1}$$

where

$$a_1 = \frac{1}{\lambda_1} + \frac{1}{\lambda_2} + \ldots + \frac{1}{\lambda_n}, \quad a_2 = \frac{1}{\lambda_2} + \ldots + \frac{1}{\lambda_n}, \quad a_n = \frac{1}{\lambda_n}. \tag{E.2}$$

Note that

$$a_1 > a_2 > \ldots > a_n \Rightarrow \beta \leq \frac{a_1}{a_n}, \tag{E.3}$$

where equality happens when all k_is are zero except k_n. Using the same methodology one can see that minimum of β happens when all the rates are zero except k_1. The minimum value of β is one.

Appendix F

Cell-to-cell variability in synchronized cells

Statistical moments conditioned on the cell cycle stage C_i can be obtained using

$$\overline{\langle x|c_i\rangle} = \frac{\overline{\langle xc_i\rangle}}{\overline{\langle c_i\rangle}}, \ \overline{\langle x^2|c_i\rangle} = \frac{\overline{\langle x^2c_i\rangle}}{\overline{\langle c_i\rangle}}. \tag{F.1}$$

In order to calculate stochastic variation in protein levels in synchronized cells we need to calculate $\langle x^2c_i\rangle$

$$\frac{d\langle x^2c_1\rangle}{dt} = 2k_1\langle B\rangle\langle xc_1\rangle + k_1\langle B^2\rangle\langle c_1\rangle + \frac{1}{4}\alpha\lambda_n \langle xc_n\rangle + \frac{\lambda_n}{4}\left\langle x^2c_n\right\rangle - \lambda_1\langle x^2c_1\rangle, \tag{F.2a}$$

$$\frac{d\langle x^2c_i\rangle}{dt} = 2k_i\langle B\rangle\langle xc_i\rangle + k_i\langle B^2\rangle\langle c_i\rangle - \lambda_i\langle x^2c_i\rangle + \lambda_{i-1}\langle x^2c_{(i-1)}\rangle, \ i = \{2,\ldots,i\}. \tag{F.2b}$$

In order to calculate $\langle x^2c_n\rangle$ we introduce the moment dynamics of $\langle x^2\rangle$

$$\frac{d\langle x^2\rangle}{dt} = 2\left(\sum_{i=1}^{n} k_i\langle xc_i\rangle\right)\langle B\rangle + \left(\sum_{i=1}^{n} k_i\langle c_i\rangle\right)\langle B^2\rangle + \frac{1}{4}\alpha\lambda_n\langle xc_n\rangle - \frac{3}{4}\lambda_n\langle x^2c_n\rangle, \tag{F.3}$$

hence in steady-state

$$\overline{\langle x^2c_n\rangle} = \frac{8}{3\lambda_n}\frac{\left(\sum_{j=1}^{n}\frac{k_j}{\lambda_j}\right)^2 + \sum_{i=1}^{n}\frac{k_i}{\lambda_i}\sum_{j=1}^{i}\frac{k_j}{\lambda_j}}{\sum_{j=1}^{n}\frac{1}{\lambda_j}}\langle B\rangle + \frac{4}{3\lambda_n}\frac{\sum_{j=1}^{n}\frac{k_j}{\lambda_j}}{\sum_{j=1}^{n}\frac{1}{\lambda_j}}\langle B^2\rangle + \frac{2\alpha}{3\lambda_n}\frac{\sum_{j=1}^{n}\frac{k_j}{\lambda_j}}{\sum_{j=1}^{n}\frac{1}{\lambda_j}}\langle B\rangle. \tag{F.4}$$

By using a similar process used in the previous section we calculate moments $\overline{\langle x^2 c_i \rangle}$

$$\overline{\langle x^2 c_i \rangle} = \frac{2}{3\lambda_i} \frac{\left(\sum_{j=1}^{n} \frac{k_j}{\lambda_j}\right)^2}{\sum_{j=1}^{n} \frac{1}{\lambda_j}} \langle B \rangle + \frac{2}{3\lambda_i} \frac{\sum_{i=1}^{n} \frac{k_i}{\lambda_i} \sum_{j=1}^{i} \frac{k_j}{\lambda_j}}{\sum_{j=1}^{n} \frac{1}{\lambda_j}} \langle B \rangle + \frac{2}{\lambda_i} \frac{\sum_{s=1}^{i} \frac{k_s}{\lambda_s} \sum_{j=1}^{s} \frac{k_j}{\lambda_j}}{\sum_{j=1}^{n} \frac{1}{\lambda_j}} \langle B \rangle$$
$$+ \frac{2}{\lambda_i} \frac{\sum_{j=1}^{i} \frac{k_j}{\lambda_j} \sum_{i=1}^{n} \frac{k_i}{\lambda_i}}{\sum_{j=1}^{n} \frac{1}{\lambda_j}} \langle B \rangle + \frac{1}{3\lambda_i} \frac{\sum_{j=1}^{n} \frac{k_j}{\lambda_j}}{\sum_{j=1}^{n} \frac{1}{\lambda_j}} \langle B^2 \rangle + \frac{1}{\lambda_i} \frac{\sum_{j=1}^{i} \frac{k_j}{\lambda_j}}{\sum_{j=1}^{n} \frac{1}{\lambda_j}} \langle B^2 \rangle + \frac{2\alpha}{3\lambda_i} \frac{\sum_{j=1}^{n} \frac{k_j}{\lambda_j}}{\sum_{j=1}^{n} \frac{1}{\lambda_j}} \langle B \rangle. \tag{F.5}$$

By having $\overline{\langle x c_i \rangle}$ and $\overline{\langle x^2 c_i \rangle}$ from (3.5) and (F.5), we can calculate the mean and the noise in synchronized cells. Using (F.1) yields the following conditional mean

$$\langle x | c_i = 1 \rangle = \left(\sum_{j=1}^{n} \frac{k_j}{\lambda_j} + \sum_{j=1}^{i} \frac{k_j}{\lambda_j} \right) \langle B \rangle. \tag{F.6}$$

Further, the protein variability level given that cells are in stage C_i is given by

$$\eta|_{c_i=1} = \underbrace{\left(\frac{2}{3} \sum_{j=1}^{n} \left(\frac{1}{\lambda_j} \right) \frac{\sum_{i=1}^{n} \frac{k_i}{\lambda_i} \sum_{j=1}^{i} \frac{k_j}{\lambda_j} + 3 \sum_{s=1}^{i} \frac{k_s}{\lambda_s} \sum_{j=1}^{s} \frac{k_j}{\lambda_j} + \left(\sum_{j=1}^{i} \frac{k_j}{\lambda_j} \right)^2 (3+\beta_c)\beta_c}{\left(\sum_{j=1}^{i} \frac{k_j}{\lambda_j} \right)^2 (1+\beta_c)^2} - 1 \right)}_{\text{Cell cycle variations}}$$

$$+ \underbrace{\left(\frac{1}{3} + \frac{2}{3} \frac{1}{1+\beta_c} \right) \frac{\langle B^2 \rangle}{\langle B \rangle} \frac{1}{\langle x | c_i \rangle}}_{\text{Burst synthesis variability}} + \underbrace{\frac{2\alpha}{3} \frac{\beta_c}{1+\beta_c} \frac{1}{\langle x | c_i \rangle}}_{\text{Partitioning errors}}, \tag{F.7}$$

where

$$\beta_c = \frac{\sum_{j=1}^{n} \frac{k_j}{\lambda_j}}{\sum_{j=1}^{i} \frac{k_j}{\lambda_j}}. \tag{F.8}$$

Appendix G

Incorporating cell-cycle times correlations, promoter switching, and mRNA dynamics in the model

In the main article we assumed independent cell-cycle times, and instantaneous transcriptional and translational bursts. Here we relax these assumptions and study how the noise in the protein level is changed. In order to include promoter switching and mRNA dynamics we consider that during the cell cycle, a gene becomes active (ON) and after an exponentially distributed time interval it becomes inactive (OFF) with a rate k_{off}. The gene spends another exponentially distributed time interval in OFF state before it becomes active again with a rate k_{on}. The mRNA molecules are produced from active states of gene with rate k_m, and they degrade with a rate γ_m per mRNA molecule. Finally, stable proteins are translated from mRNA with rate k_x. Let $g(t)$ be indicator function of gene, $g(t) = 1$ ($g(t) = 0$) means gene is ON (OFF), then the model contains the following stochastic events during the cell cycle

$$\text{Gene activation:} \quad g(t) \xmapsto{k_{on}(1-g(t))} g(t) + 1, \tag{G.1a}$$

$$\text{Gene deactivation:} \quad g(t) \xmapsto{k_{off}g(t)} g(t) - 1, \tag{G.1b}$$

$$\text{mRNA production:} \quad m(t) \xmapsto{k_m g(t)} m(t) + 1, \tag{G.1c}$$

$$\text{mRNA degradation:} \quad m(t) \xmapsto{\gamma_m m(t)} m(t) - 1, \tag{G.1d}$$

$$\text{Protein production:} \quad x(t) \xmapsto{k_x m(t)} x(t) + 1, \tag{G.1e}$$

where $m(t)$ and $p(t)$ denote mRNA and protein population levels at time t, respectively. At the end of cell cycle, division occurs and mRNA and protein molecules are partitioned in daughter cells binomially. After each division we select a new cell-cycle time which is correlated to previous cell-cycle times. We add correlation to the new cell-cycle time $T_i,\ i \in \mathbb{N}$, by assuming that it is connected to previous cell-cycle time through an Auto Regressive (AR) process

$$T_i = T_0 + \phi T_{i-1} + \eta_i, \tag{G.2}$$

where η_is are independent and identical normally distributed random variables $\eta_i \sim \mathcal{N}(0, \sigma_\eta)$, T_0 is a constant, and $|\phi| < 1$. For this model the mean and variance of cell-cycle time is

$$\langle T_i \rangle = \frac{T_0}{1-\phi},\ i \in \mathbb{N}, \qquad Var(T_i) = \frac{\sigma_\eta^2}{1-\phi^2}. \tag{G.3}$$

Further the cross correlation between two cell cycles which are i cycles apart is ϕ^i.

In the case of transcriptional bursting, burst frequency is gene activation rate, i.e., k_{on} in this model. Hence here we assumed that k_{on} is a function of cell-cycle time. We investigate two scenarios 1) constant gene activation rate 2) synthesis at the end of cell cycle. For constant k_{on}, gene switches between ON and OFF states through the cell cycle. In the synthesis at the end of cell cycle, we assume that for 75% of T_i gene is OFF and $k_{on} = 0$. In the last 25% of cell cycle time switching occurs and k_{on} is non zero. Further transcriptional bursting is the limit of large k_{off} and small k_{on}, i.e., genes is OFF most of the time. Here we consider that gene is active for 20% of the cell-cycle time. Further we analyzed the system in both fast and slow switching switching rates.

We use another model in which protein production is modeled deterministic through-

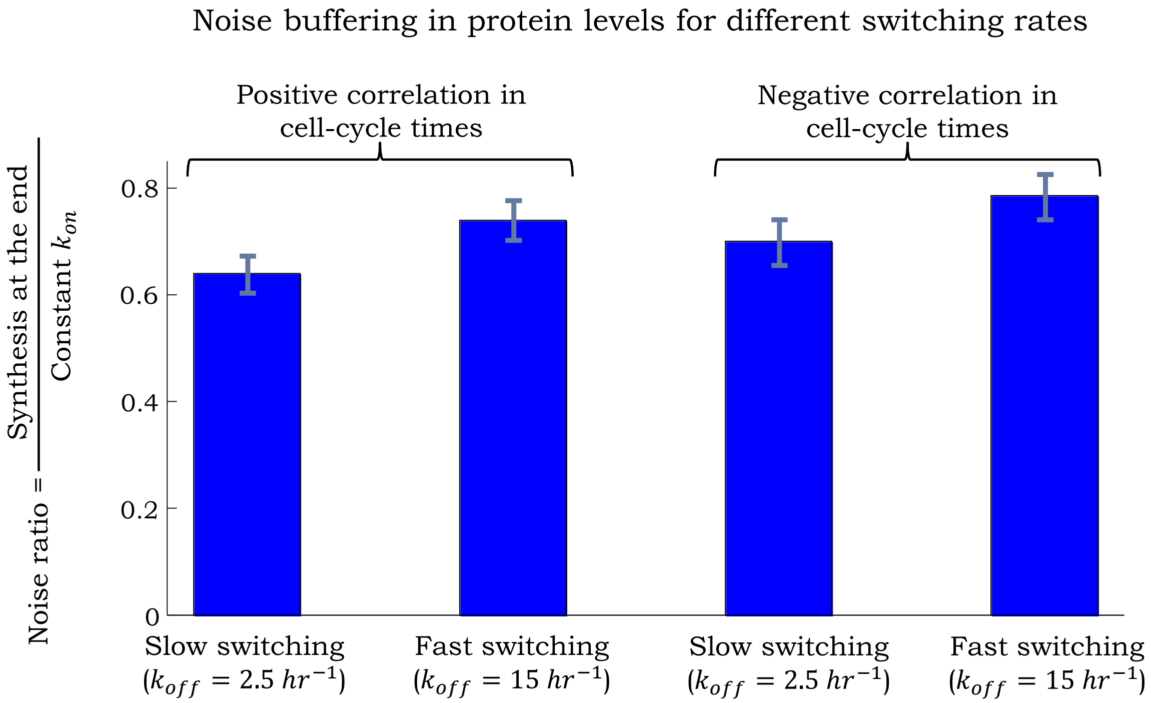

Figure S1: **Protein synthesis at the end of cell cycle reduces noise contributed from expression in the limit of slow switching rates and presence of correlated cell cycle times.** Noise ratio less than 1 indicates syntheis at the end reduces the noise in comparison with constant production. Noise ratio is less than one for different switching rates and correlation values. For this plot we have assumed both negative correlation of -0.25 [5] and positive correlation of 0.25 between successive cell-cycle times, Mean cell-cycle time is 2 $hours$ and noise in cell-cycle times is $CV_T^2 = 0.05$. The mRNA production rate is $k_m = 50hr^{-1}$ and mRNA molecules degrade with rate $\gamma_m = 5hr^{-1}$. Protein molecules are translated from mRNA with a rate $k_p = 25hr^{-1}$. Gene activation rate k_{on} is adjusted to keep mean of protein equal to 150 molecules for all cases. The error bars obtained via bootstrapping by using $20,000$ Monte Carlo simulations.

out the cell cycle

$$\frac{dg(t)}{dt} = k_{on} - (k_{on} + k_{off})g(t), \ \frac{dm(t)}{dt} = k_m g(t) - \gamma_m m(t), \ \frac{dp(t)}{dt} = k_p m(t). \tag{G.4}$$

In the time of division mRNA and proteins are partitioned based on a beta distribution which is the continuous counter part of binomial distribution. The difference between

noise levels of these two models give the noise from stochastic expression.

We numerically investigate the models described in (G.1) and (G.4) for correlated cell-cycle times in (G.2). Figure S1 shows the simulation results obtained from $20,000$ Monte Carlo simulations for different switching rates and correlation values. From equation (4.7) in the main article we know that synthesis at the end of cell cycle reduces noise in comparison with constant synthesis. However equation (4.7) obtained for the bursty expression model which is an approximation in the limit of fast switching. Moreover in order to derive (4.7), cell cycle time are assumed to be independent. Simulation results reveal that in the presence of correlated cell-cycle times and by taking into account dynamics of gene and mRNA, synthesis at the end still reduces the noise contribution from stochastic synthesis. This reduction happens even when gene is active for relatively long time, switching is slow, dynamics of mRNA is included and cell-cycle times are correlated. In summary our analysis reveals that perturbing the assumptions made in this paper to obtain analytic solutions are not changing the fact that synthesis at the end of cell cycle leads to buffering noise contributed from stochastic expression.

Bibliography

1. D. A. McQuarrie, “Stochastic approach to chemical kinetics,” *Journal of Applied Probability*, vol. 4, pp. 413–478, 1967.

2. D. T. Gillespie, “Approximate accelerated stochastic simulation of chemically reacting systems,” *Journal of Chemical Physics*, vol. 115, pp. 1716–1733, 2001.

3. J. P. Hespanha and A. Singh, “Stochastic models for chemically reacting systems using polynomial stochastic hybrid systems,” *International Journal of Robust and Nonlinear Control*, vol. 15, pp. 669–689, 2005.

4. A. Singh and M. Soltani, “Quantifying intrinsic and extrinsic variability in stochastic gene expression models,” *PLOS ONE*, vol. 8, p. e84301, 2013.

5. S. Taheri-Araghi, S. Bradde, J. T. Sauls, N. S. Hill, P. A. Levin, J. Paulsson, M. Vergassola, and S. Jun, “Cell-size control and homeostasis in bacteria,” *Current Biology*, vol. 25, pp. 385–391, 2015.
